# A novel single-port laparoscopic operation for colorectal cancer with transanal specimen extraction: a comparative study

**DOI:** 10.1186/1471-2482-15-10

**Published:** 2015-01-30

**Authors:** Say-June Kim, Byung-Jo Choi, Sang Chul Lee

**Affiliations:** Department of Surgery, Daejeon St. Mary’s Hospital, The Catholic University of Korea, Daeheung-dong 520-2, Daejeon, Jung-gu, Republic of Korea

**Keywords:** Colorectal cancer, Laparoscopy, Sigmoidectomy, Single-port laparoscopic surgery, Specimen extraction

## Abstract

**Background:**

Extension of a single incision for the purpose of specimen extraction in single-port laparoscopic surgery (SPLS) can undermine the merits of SPLS, either by hurting cosmesis or by increasing wound morbidity.

**Methods:**

We retrospectively analyzed the clinical outcomes of patients undergoing SPLS sigmoidectomy, either with transanal specimen extraction (TASE, n = 15) or transumbilical specimen extraction (TUSE, n = 68), for colorectal cancer between March 2009 and March 2013. The inclusion criterion was a tumor diameter of ≤ 5 cm. The median follow-up was 93 months (range 13 – 149).

**Results:**

Most of intraoperative and postoperative variables were comparable between the two groups, except for lengthening of operation time in TASE (287 ± 87 min vs. 226 ± 78 min, *P* = 0.011). TUSE did not lengthen the duration of postoperative recovery, hospital stay, or pain, or increase the incidence of postoperative complications. Whereas TUSE showed 8.8% (6/68) of wound-related complications, TASE did not show wound-related complications during follow-up period (*P* = 0.586).

**Conclusion:**

With the exception of a prolonged operation time, TASE showed equivalent surgical outcomes as TUSE in SPLS sigmoidectomy. Thus, the implement of TASE is expected to provide one way of reducing wound-related complications in SPLS in patients with a tumor diameter of ≤5 cm.

## Background

In the era of laparoscopy, pioneering surgeons continue to attempt to reduce the size and number of incision(s) in order to maximize the benefits of minimally invasive surgery. The size and number of incision(s) is important because these parameters are closely related to the risk of various postoperative sequelae, such as pain, infection, injury to the vessels and nerves of the abdominal wall, and incisional hernia [1–3]. In this respect, the introduction of single-port laparoscopic surgery (SPLS) has raised the possibility of overcoming, or at least effectively reducing, wound-related morbidity. SPLS does dramatically reduce the number of surgical wounds. However, when it is necessary to extract a bulky specimen, such as the liver, spleen, or an intestinal segment, a corresponding incision size is still required, which simultaneously compromises the benefits of SPLS and increases wound morbidity. Therefore, it is essential to find a method for reducing the incision size required for specimen extraction.

The pursuit of a surgical technique that involves no external wound has led to the development of natural orifice transluminal endoscopic surgery (NOTES) [4–6]. The fundamental concept of NOTES is to reach the operative field through a natural orifice, such as the oral cavity, vagina, or anal canal, thereby circumventing the abdominal wall. Until now, most attempts at NOTES are still in the preclinical trial stage because of technical difficulties [7–9]. However, this method has inspired laparoscopic surgeons to borrow the basic concept of NOTES and adapt it for laparoscopic surgery [8, 10]; consequently, hybrid laparoscopic techniques, combining laparoscopic surgical techniques with natural orifice specimen extraction (NOSE), have been developed [11–13].

NOSE can be performed via the stomach, colorectum, anus, and vagina. In colectomies, the preferred specimen extraction site is the anus because the colectomy procedure naturally makes way for specimen extraction without an additional intraorgan incision [14–16]. However, the feasibility and safety of transanal NOSE in SPLS has not yet been determined, and to the best of our knowledge, no comparative studies have been performed thus far. Therefore, we attempted to determine the role of transanal specimen extraction (TASE) by comparing its surgical outcomes with those of transumbilical specimen extraction (TUSE) in single-port anterior resection (AR) or low anterior resection (LAR) for colorectal cancer.

## Methods

### Study design and data collection

**Figure 1:**
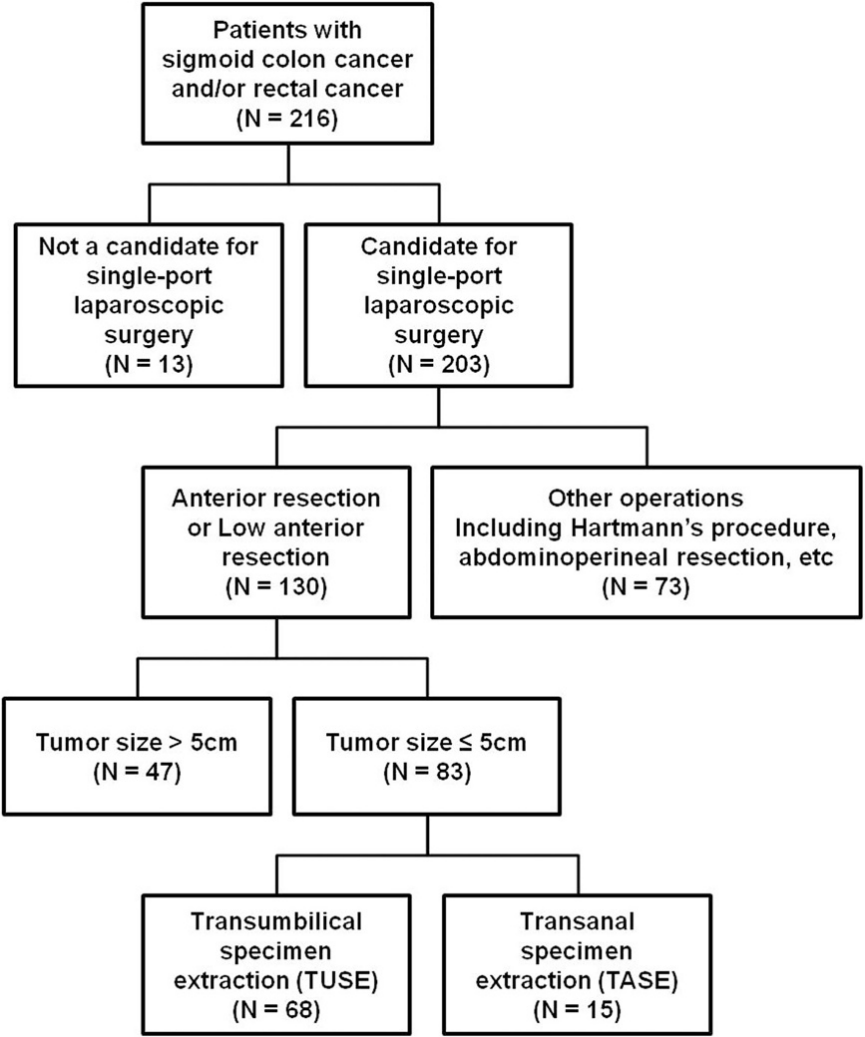
**Patient allocation.**

The prospectively collected records of patients who underwent surgery for sigmoid colon cancer and/or rectal cancer at Daejeon St. Mary’s Hospital, the Catholic University of Korea, between March 2009 and March 2013, were reviewed retrospectively (Figure 1). A total of 216 patients were enrolled at this stage. During this period, SPLS was first attempted in colorectal cancer patients eligible for operation (i.e., those who did not have advanced local disease [tumor size > 10 cm on preoperative evaluation], unresectable metastatic lesions, an American Society of Anesthesiologists’ physical status classification of IV or V, or severe medical illness). History of prior laparotomy and/or the presence of acute bowel obstruction did not preclude SPLS. Consequently, we identified 203 patients who had undergone SPLS for sigmoid colon cancer and/or rectal cancer. These patients had been treated by various operative methods via a single port, including AR, LAR, abdominoperineal resection, Hartmann’s procedure, total colectomy, transanal endoluminal laparoscopic surgery, and transabdominal transanal resection of the sigmoid colon. Of these various operative methods, single-port AR or LAR were indicated when the patients were judged to have no other colonic lesion(s) outside of the sigmoid colon and/or rectum; when primary colonic or colorectal anastomosis after sigmoidectomy seemed possible; or when the lesion was located sufficiently far from the anal verge so as to preserve the rectal sphincter and permit safe end-to-end anastomosis (EEA) stapler application. Consequently, 130 patients who had undergone single-port AR or LAR were identified. After colectomy in single-port AR or LAR, TUSE or TASE was performed to retrieve specimens. TASE was selectively performed when the tumor diameter appeared to be 5 cm or less in the preoperative evaluation and the rectal canal could be sufficiently dilated up to 5 cm with an anal trocar. Therefore, to provide a balanced comparison, we selected 83 patients in whom the tumor diameter was 5 cm or less from the patient population (n = 130), and clinical outcomes were compared between the TUSE group (n = 68) and TASE group (n = 15). This study was approved by the ethics committee at our institution (Institutional Review Board of Daejeon St. Mary’s hospital, College of Medicine, the Catholic University of Korea, IRB code: DC13RISI0079). Electronic medical records, including radiology and pathology reports, of all patients in each group were deliberately reviewed to ensure accuracy. The median follow-up was 93 months (range 13 – 149).

A complication was defined as the occurrence of any adverse event before discharge. Postoperative complications were classified as described by Clavien and colleagues [17]. Delayed gastric emptying was defined as when a nasogastric tube was required for ≥ 4 postoperative days or if its reinsertion was required, or when the patient remained intolerant to solid diet by postoperative day 7. Urinary retention was defined as when the patient could not pass urine within 12 h after removal of the urinary catheter. Operative time was measured from the time of initial skin incision to completion of wound closure, based on documentation by the anesthesiologist. Pathological margins were determined by two pathologists (Kim JO, Lee JU) based on formalin-fixed specimens. Staging was based on the 6th edition of the American Joint Committee on Cancer manual [18].

### Operative technique

**Figure 2:**
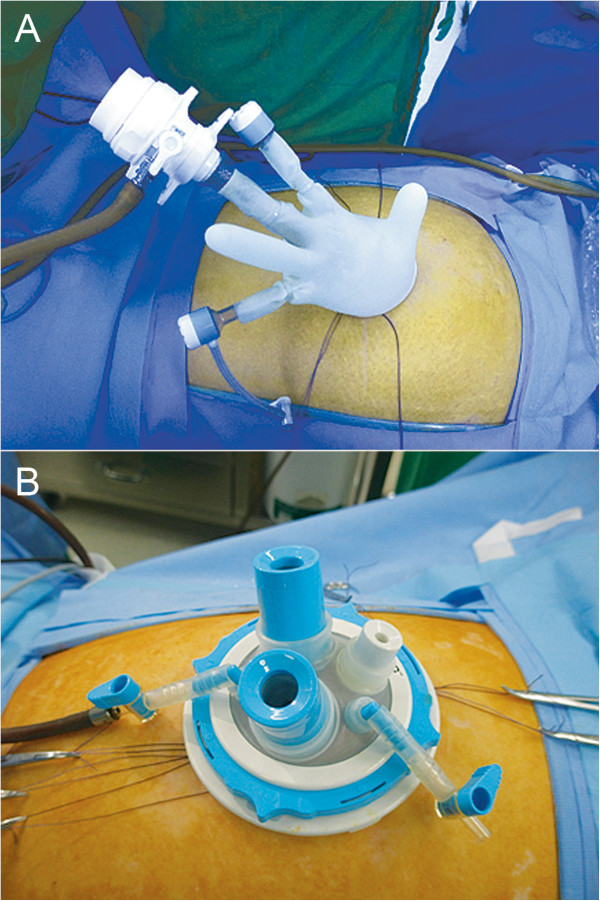
**Placement of single-ports in the umbilicus. A** Placement of homemade glove port composed of a wound retractor (ALEX wound retractor; XS, USA), a surgical glove, and two pipes (threaded cannulas and seals 5 mm; Applied Medical, USA). **B** Placement of a commercially ready-made single port (OCTO port; Dalim, Korea).

Under general anesthesia, the patient was placed in the modified lithotomy position. The operating surgeon and camera operator were positioned on the right side of the patient, and the first assistant was positioned on the left side. Usually, a 1.5- to 2.0-cm vertical incision was made at the umbilicus. Initially, we designed and used a single-port system composed of a wound retractor (ALEX wound retractor; XS, USA), a surgical glove, 2 pipes (5-mm threaded cannulas and seals; Applied Medical, USA), and a trocar (Xcel 12 mm; Ethicon, USA) (Figure 2A). Later, we replaced this system with a commercially available ready-made single port system (OCTO port; Dalim, Korea) that contains a 5-mm trocar and two 12-mm trocars (Figure 2B). After mobilization of the sigmoid colon in a medial-to-lateral fashion, we incised the retroperitoneum between the sacral promontory and aortic bifurcation while taking care to preserve the hypogastric nerve plexus. The inferior mesenteric artery and vein were then identified and divided, respectively. Next, the splenic flexure was mobilized, if necessary. The proximal rectum was dissected free, starting from the mesorectum. After the proximal and distal resection margins of the tumor-bearing segment had been determined and fully mobilized, we divided the sigmoid mesentery with a vessel-sealing energy device (Ligasure, Covidien, USA). Thereafter, the colon and proximal rectum were tied with a nonabsorbable suture (Ethibond EXCEL™ Polyester suture, Ethicon, USA) to isolate the specimen and to minimize soiling.

Total mesorectal excision (TME) was performed in all cases of rectal cancer. Before TME, we ensured the visual field by elevating the peritoneal fold (male) or the uterus (female) with an intracorporeal stitch. Anterior dissection of TME widened the gap between the anterior rectal wall and the Denonvillier’s fascia in men or the posterior vaginal wall in women. In addition, posterior and lateral dissection of TME reached the level of the puborectalis muscle. Thereafter, the proximal and distal ends of the lesion were completely enclosed with nylon tape to prevent cancer dissemination. The following steps differed according to the method of specimen extraction (TUSE or TASE).

**Figure 3:**
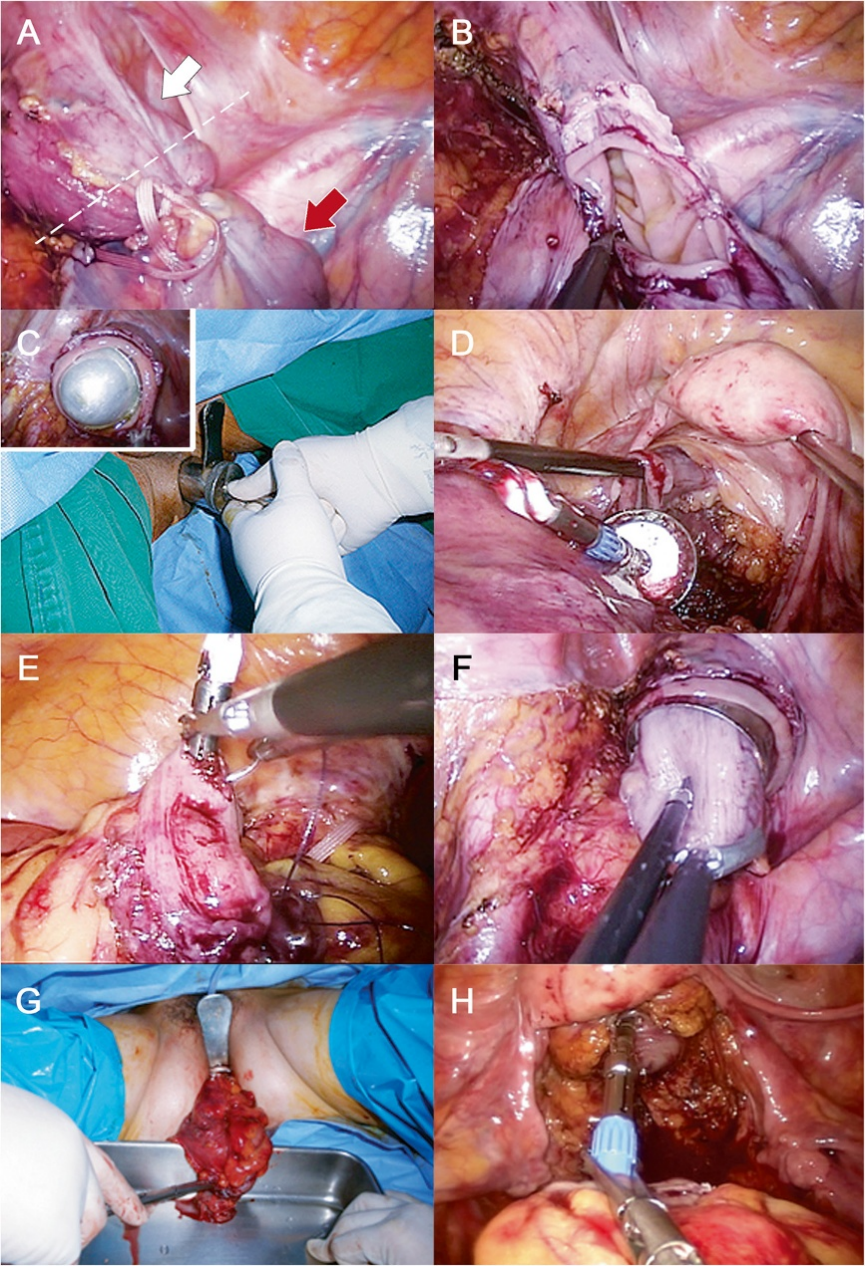
**Operative illustrations showing single-port laparoscopic colectomy with transanal specimen extraction (TASE).** After dissection, both ends of the tumor-bearing segment were bound with tape, and the proximal end was divided by End-GIA. **A** The distal end of the tumor-bearing segment was identified. The white arrow indicates the direction to the rectum. The red arrow indicates the tumor-bearing segment. The dotted line indicates the planed resection line. **B** The tumor-bearing segment was completely resected by dividing the distal end. **C** An anal trocar was entered into the pelvic cavity via the anus. **D** An anvil with an anchor suture was entered into the pelvic cavity through the anal trocar. **E** The anvil was introduced into the remaining colon and was fixed by purse-string suture. **F, G** Thereafter, the specimen was retrieved through the anal trocar. **H** Lastly, end-to-end colorectal anastomosis was performed with a transanally inserted circular stapler (EEA 28 mm or 31 mm; Ethicon, USA).

**Figure 4:**
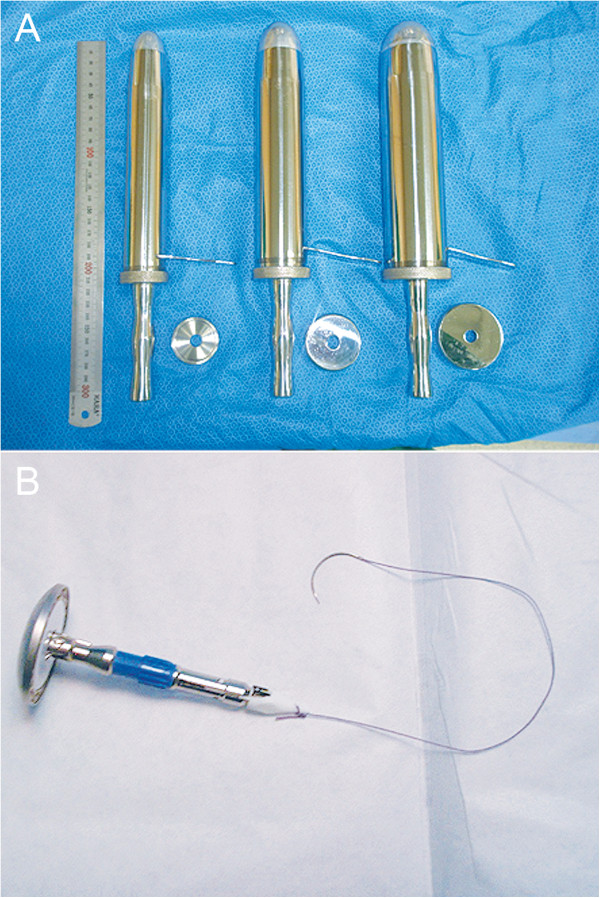
**Prerequisites of transanal specimen extraction using our method. A** Anal trocars. They are metal cylinders with a range of diameters (3–6 cm) that are designed for specimen extraction via the anal canal. **B** An anvil with an anchor suture. The tip of the anvil was anchored with the aim of facilitating an intracorporeal purse-string suture.

In patients in whom TUSE was performed, the distal end of the tumor-bearing segment was divided with a stapler (Endo-gastrointestinal anastomosis [GIA] Green cartilage; Covidien, USA). The tumor-bearing segment was subsequently delivered extracorporeally through the umbilical wound after optimal extension of the skin incision. Extracorporeally, the proximal end of the tumor-bearing segment was divided, and an anvil for EEA was inserted in the remaining colon. After returning the bowel to the abdominal cavity, end-to-end colorectal anastomosis was performed with a transanally inserted circular stapler (EEA 28 mm or 31 mm; Ethicon, USA).In patients in whom TASE was performed, both the proximal and distal ends of the tumor-bearing segment were divided with a stapler (Endo-GIA Green cartilage; Covidien, USA) and endoscissors, respectively (Figure 3). The anal canal was then thoroughly cleansed by irrigation with povidone-mixed saline solution. Next, an anal trocar (i.e., a metal cylinder with a diameter of 3–6 cm) was placed through the anal canal (Figure 4A). Using a series of anal trocars, the anal canal was gradually dilated to prevent injury to the rectal wall and anal sphincter due to excessive pressure. To facilitate a purse string suture, we designed an anvil with an anchoring suture (Figure 4B). The anvil was entered into the pelvic cavity via the anal trocar and then introduced in the remaining colon. The anvil was put in the pelvic cavity via the open anal canal and was inserted and fixed in the remaining colon using intracorporeal purse string suture and Endo-GIA stapling. The specimen was extracted smoothly through the anal canal. Thereafter, the open distal rectal stump was sutured with an Endo-GIA stapler or by hand-sewn sutures. Colorectal anastomosis was completed using the transanally inserted circular stapler.

Regardless of the method of specimen extraction, a Jackson-Pratt drain was inserted through the single-port incision site, as needed.

### Postoperative care

Postoperative diet was initiated and advanced as previously described [19]. Postoperative pain was first managed by patient-controlled administration of intravenous fentanyl citrate, and additional intravenous medications for pain control were given as needed. The urinary catheter was typically removed on postoperative day 1.

### Statistical analysis

The results are presented as the mean ± standard deviation and/or median (range). Continuous variables were compared with the Mann–Whitney U-test or independent t-test, depending on the normality of the quantitative variables. Categorical and ordinal variables were compared with the chi-square test. Statistical analysis was performed with SPSS version 15.0 (SPSS Inc., Chicago, IL, USA). A p value < 0.05 was considered statistically significant.

## Results

### Basal characteristics and pathological comparisons

This study included 83 patients (47 men, 36 women), comprising the TUSE group (n = 68) and TASE group (n = 15). The median age was 66 years (range, 38–82 years), and the median body mass index was 23.2 (16.2–30.3). Of these patients, 52 patients (62.7%) had sigmoid colon cancer (including cancers of the rectosigmoid junction), and 31 patients (37.3%) had rectal cancer. Single-port AR was performed in 43 patients (51.8%), and single-port LAR was performed in 40 patients (48.2%). The baseline demographics and patient characteristics between these two groups were compared (Table 1). The two groups were similar in terms of baseline characteristics, such as age, sex, body mass index, or Charlson comorbidity index. There were also no differences in locations of lesions and the operative method (AR or LAR) between the two groups.

**Table 1:** **Patient demographics and baseline characteristics**

Patient characteristics	Total patients (n = 83)	TUSE (n = 68)	TASE (n = 15)	P-value
Age (years)				0.442
Median (range)	66.0 (38.0–82.0)	66.0 (38.0–82.0)	65.0 (50.0–75.0)	
Mean ± SD	63.9 ± 10.3	64.3 ± 11.0	62.0 ± 8.3	
Sex, n (%)				1.000
Men	47 (56.6)	38 (55.9)	6 (40.0)	
Women	36 (43.4)	30 (44.1)	9 (60.0)	
Body-mass index, kg/m^2^ (%)				0.281
Median (range)	23.2 (16.2–30.3)	23.5 (16.2–30.3)	22.0 (18.7–26.7)	
Mean ± SD	23.0 ± 2.9	23.2 ± 3.0	22.3 ± 2.4	
Charlson comorbidity index, n (%)				0.091
Charlson index = 0	37 (44.6)	28 (41.2)	10 (66.7)	
Charlson index > 0	46 (55.4)	40 (58.8)	5 (33.3)	
The location of lesion				0.151
Sigmoid colon (including the rectosigmoid junction)	52 (62.7)	40 (58.8)	12 (80.0)	
Rectum	31 (37.3)	28 (41.2)	3 (20.0)	
Operative method				0.259
Anterior resection	43 (51.8)	33 (48.5)	10 (66.7)	
Low anterior resection	40 (48.2)	35 (51.5)	5 (33.3)	

*Abbreviations*: *SD* standard deviation, *TASE* transanal specimen extraction, *TUSE* transumbilical specimen extraction.

### Comparison of intraoperative and pathological variables

**Figure 5:**
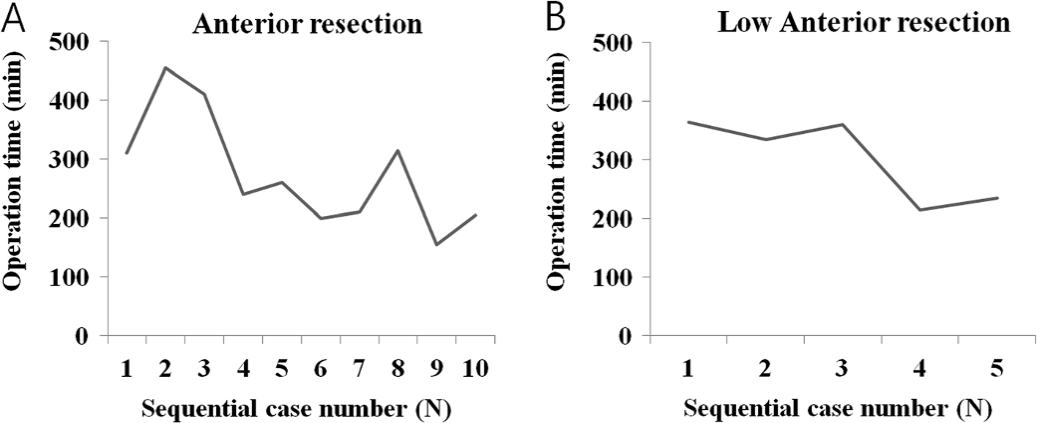
**The changes in the operative times of the TASE (transanal specimen extraction) group following single-port anterior resection (A) and low anterior resection (B).**

Table 2 shows the comparison of operative details and pathological outcomes between the TUSE and TASE groups. TASE resulted in a longer operative time than did TUSE (285 ± 87 min vs. 226.0 ± 78.0 min; p = 0.011). Thereafter, we illustrated individual operation times of TASE cases over time, according to the operative method (AR or LAR) (Figure 5). The sequential operation time of the TASE group appeared to decrease over time, reflecting learning processes.

**Table 2:** **Data related to operative details and tumor pathology**

Characteristics	Total patients (n = 83)	TUSE (n = 68)	TASE (n = 15)	P-value
Overall operative time (min)				0.011
Median (range)	215 (95–455)	215 (95–455)	260 (155–455)	
Mean ± SD	237 ± 82	226 ± 78	287 ± 87	
Estimated blood loss, mL				0.884
Median (range)	200 (20–1000)	200 (20–1000)	300 (50–750)	
Mean ± SD	282 ± 191	279 ± 196	287 ± 171	
PRC transfused patients, n (%)	5 (6.0)	3 (4.4)	2 (13.7)	0.220
Intraoperative complications, n (%)				1.000
Vascular injury	1 (1.2)	1 (1.5)	0	
Major serosal tearing	2 (2.4)	2 (2.9)	0	
Total (%)	3 (3.6)	3 (4.4)	0 (0.0)	
Duration of drain installation, days				0.371
Median (range)	4 (0–14)	4 (0–14)	4 (0–9)	
Mean ± SD	4.1 ± 2.5	4.4 ± 2.9	3.6 ± 1.9	
Tumor differentiation, n (%)				0.083
Well differentiated	3 (3.6)	1 (1.5)	2 (13.3)	
Moderately differentiated	80 (96.4)	67 (98.5)	13 (86.7)	
Poorly differentiated	0 (0.0)	0 (0.0)	0 (0.0)	
Tumor depth (T classification), n (%)				0.013
T1	16 (19.3)	11 (16.2)	5 (33.3)	
T2	22 (26.5)	15 (22.0)	7 (46.7)	
T3	45 (54.2)	42 (61.8)	3 (20.0)	
Lymph node metastasis, n (%)				0.331
No	81 (97.6)	67 (98.5)	14 (93.3)	
Yes	2 (2.4)	1 (1.5)	1 (6.7)	
Tumor stage, n (%)				0.062
I	24 (28.9)	16 (23.5)	8 (53.3)	
II	19 (22.9)	17 (25.0)	2 (13.3)	
III	38 (45.8)	34 (50.0)	4 (26.7)	
IV	2 (2.4)	1 (1.5)	1 (6.7)	
Largest tumor diameter (cm)				0.220
Median (range)	4.0 (0.2–5.0)	4.0 (0.3–5.0)	3.0 (2.0–5.0)	
Mean ± SD	3.3 ± 1.4	3.4 ± 1.3	3.0 ± 1.8	
Lymph nodes in resected specimen				0.785
Median (range)	17.0 (0–49)	17.0 (0–49)	18 (6–41)	
Mean ± SD	17.1 ± 9.3	17.0 ± 9.4	17.7 ± 9.6	
Proximal margin (cm)				0.744
Median (range)	7.0 (3.0–105.0)	6.0 (3–105)	8.0 (4.0–20.0)	
Mean ± SD	10.0 ± 12.2	9.4 ± 13.2	9.5 ± 4.6	
Distal margin (cm)				0.359
Median (range)	5.0 (2.0–37.0)	5.4 (3–37)	5.0 (4.0–12.5)	
Mean ± SD	7.0 ± 5.0	7.2 ± 5.2	6.2 ± 2.7	
Perineural invasion, n (%)				0.749
No	61 (73.5)	49 (72.1)	12 (80.0)	
Yes	22 (26.5)	19 (27.9)	3 (20.0)	
Lymphovascular invasion, n (%)				0.007
No	16 (19.3)	9 (13.2)	7 (46.7)	
Yes	67 (80.7)	59 (86.8)	8 (53.3)	

*Abbreviations*: *PRC* packed red blood cells, *SD* standard deviation, *TASE* transanal specimen extraction, *TUSE* transumbilical specimen extraction.

The estimated blood losses and the amount of packed red cell transfusion was not significantly different between the two groups. The incidence of intraoperative complications was also not significantly different. The median largest tumor diameters in the TUSE and TASE groups were 4.0 (0.3–5.0) cm and 3.0 (2.0–5.0) cm, respectively (p = 0.220).

Next, pathological outcomes were compared. Several parameters seemed to include more advanced pathologies in the TUSE group than in the TASE group, such as tumor depth (T1, T2, and T3 stages; 16.2%, 22.0%, and 61.8% in the TUSE group, respectively; 33.3%, 46.7%, and 20.0% in the TASE group, respectively; p = 0.013) and lymphovascular invasion (86.8% in the TUSE group vs. 53.3% in the TASE group; p = 0.007).

The other parameters, including tumor cell differentiation, lymph node metastasis, tumor stage, and perineural invasion, were comparable between the two groups. In addition, the two groups showed similar oncologic results, such as sufficient attainment of surgical margins and lymph nodes.

### Comparison of postoperative variables

**Figure 6:**
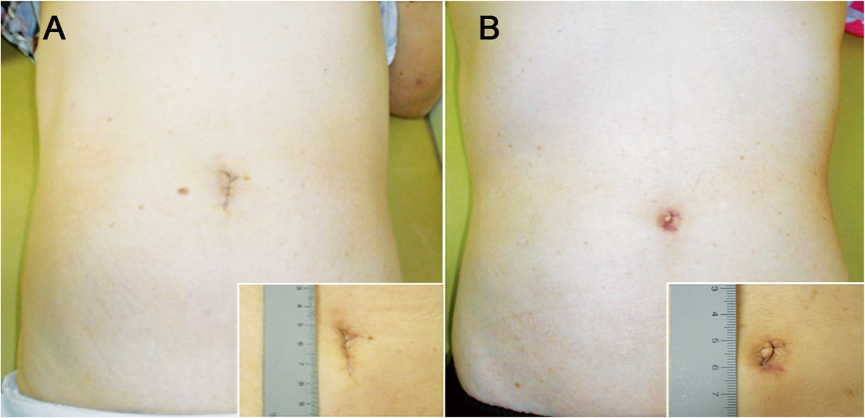
**Representative illustrations of postoperative wounds. A** Postoperative wound following transumbilical specimen extraction (TUSE). **B** Postoperative wound following transanal specimen extraction (TASE).

We then assessed and compared the postoperative recovery of gastrointestinal function, which was reflected by the intervals to first flatus, to free oral fluids, and to solid diet (Table 3). The two groups showed comparable functional recovery. The frequencies of narcotic analgesics and total analgesics administration was not significantly different between the TUSE and TASE groups. The postoperative lengths of hospital stay were also similar. Overall, anastomotic site leakage was the most common postoperative complication (6/83, 7.2%), followed by delayed gastric emptying (n = 2), urinary retention (n = 4), and pneumonia (n = 1). There were 4 and 1 incidences of anastomotic site leakages in TUSE and TASE groups, respectively. Every incidence of anastomotic site leakage required reoperation. The extent of reoperation was varied according to the severity of anastomotic site leakage. The four cases of leakages developed in TUSE group required irrigation with diverting ileostomy (n = 2), primary closure with diverting ileostomy (n = 1), and transanal closure (n = 1), respectively. The one leakage developed in the TASE group was corrected by primary closure with diverting ileostomy. All the patients with leakage were recovered successfully after reoperation, and no mortality was occurred. There was no significant difference between TUSE and TASE groups in the overall postoperative complications (*P* = 0.196).Thereafter, we compared wound-related complications during the follow-up period. Whereas TUSE group exhibited wound seromas (n = 4), wound infection (n = 1), and umbilical hernia (n = 1), TASE group showed no wound-related complications during the follow-up period. Figure 6 shows representative illustrations of postoperative wounds with TUSE and TASE (The patients in the images have specifically provided consent to publish).

**Table 3:** **Postoperative outcomes**

Postoperative variables	TUSE (n = 68)	TASE (n = 15)	P-value
Duration prior to first flatus, day(s)			0.298
Median (range)	2.0 (1.0–5.0)	2.0 (1.0–4.0)	
Mean ± SD	1.9 ± 1.1	2.2 ± 1.0	
Durations prior to free oral fluids			0.291
Median (range)	1.0 (1.0–10.0)	3.0 (1.0–10.0)	
Mean ± SD	2.2 ± 1.8	2.8 ± 2.4	
Duration prior to solid diet, day(s)			0.403
Median (range)	2.0 (1–22)	4.0 (1–14)	
Mean ± SD	3.4 ± 3.2	4.2 ± 3.2	
Frequency of narcotic analgesics			0.297
Median (range)	1.0 (0.0–25.0)	1.0 (0.0–11.0)	
Mean ± SD	3.0 ± 4.6	2.1 ± 3.1	
Frequency of total analgesics			0.448
Median (range)	2.0 (0.0–40.0)	2.0 (0.0–11.0)	
Mean ± SD	4.0 ± 6.7	3.1 ± 3.9	
Postoperative length of stay, day(s)			0.272
Median (range)	7.0 (4.0–55.0)	6 (4–16)	
Mean ± SD	10.3 ± 9.6	7.5 ± 3.6	
Overall postoperative complications, %	14.7 (10/68)	20.0 (3/15)	0.196
Grade I			
Delayed gastric emptying	0	1	
Urinary retention	1	0	
Grade II			
Urinary retention	2	1	
Delayed gastric emptying	1	0	
Pneumonia	1	0	
Grade III			
Anastomotic site leakage	5	1	
Wound-related complications during FU period, %	8.8 (6/68)	0.0 (0/15)	0.586
Seroma	4	0	
Wound infection	1	0	
Umbilical hernia	1	0	
Mortality, %	0	0	1.00

*Abbreviations*: *FU* follow-up, *SD* standard deviation, *TASE* transanal specimen extraction, *TUSE* transumbilical specimen extraction.

## Discussion

In this study, we attempted to determine the utility of TASE by comparing it with TUSE. The process of TASE prolonged the overall operation time because of the additional detailed procedures. However, the TASE group showed similar results as the TUSE group in other parameters, such as the incidence of intraoperative and postoperative complications, postoperative gastrointestinal functional recovery, the frequency of postoperative analgesics usage, and the length of hospital stay; this reflects the safety and feasibility of the procedure. Notably, though it did not reach statistical significance, wound-related complications were lower in TASE than TUSE (0.0% vs. 8.8%, *P* = 0.586). Although the sample size was too small for definitive conclusions, these preliminary results suggest the safety and feasibility of TASE.

The NOSE technique involves specimen extraction through a natural orifice, such as the anus or vagina. There are several benefits of NOSE. Most of all, NOSE can improve cosmesis dramatically by negating wound extension for specimen extraction. In addition, NOSE can reduce wound morbidities, such as wound infection, injury to the vessels and nerves of the abdominal wall, and incisional hernia [1–3]. Moreover, NOSE theoretically reduces postoperative somatic pain at the incision site. Postoperative pain after laparoscopic surgery is determined by a combination of numerous factors, including wound size, distension-induced neuropraxia of the phrenic nerves, residual intra-abdominal gas after laparoscopy, the humidity and volume of the insufflated gas, anesthetic drugs, and sociocultural and individual factors [20]. Of these, wound size constitutes a substantial portion. In this study, the TASE group required lower doses of both narcotic analgesics and total analgesics, though the difference did not reach statistical significance. Further studies with an adequately larger patient population are necessary to determine the effects of TASE on postoperative pain.

The natural orifices commonly used for specimen extraction during colectomies are the anus (TASE) and vagina (TVSE, transvaginal specimen extraction). TASE has several advantages over TVSE, especially in colorectal surgery [12]; it can be used regardless of sex, does not require additional intraorgan incision, and is technically more feasible. In contrast, the process of TVSE is more complicated due to the anatomy of Douglas’s pouch. It was reported that protective ileostomy was required more frequently in TVSE than in TASE because of accidental intraoperative damage to the sigmoid colon and rectum [12].

There are several qualifications for the ideal method of specimen extraction. First, it should ensure patient safety from the beginning of the process throughout the postoperative period. In addition, it should not be so technically difficult as to significantly prolong the operation time. Finally, the process of specimen extraction should not offset the advantages of minimally invasive surgery. Taken together, TASE may be considered a preferred method of specimen extraction after single-port AR or LAR. TASE resulted in equivalent surgical outcomes as TUSE in terms of postoperative complications, while leaving a NOTES-like scar (≤ 2 cm). The major demerit of TASE was a longer operation time; however, considering the benefits of TASE and the trends in shortening operation times, this demerit may be easily overcome.

In our study, there was a lengthening of operation time in TASE group (260 min vs. 215 min, *P* < 0.011). So far, there have been no reports comparing the operation time between TUSE and TASE in SPLS. Meantime, applications of TASE into the conventional laparoscopic surgery have been sporadically reported. Wolthuis et al. [16] reported in a systematic analysis that TASE did not lengthen the operation time in the procedures involving left-sided laparoscopic colectomy compared with TUSE. Fuchs et al.[8] also concluded after the earlier experience of TASE in the laparoscopic surgery that the application of TASE to laparoscopic surgery was quite easy and is not a major problem for an experienced laparoscopic surgeon, indicating that TASE procedure does not require a long learning curve or the acquisition of new, specialized skills. Interestingly, in a paper comparing TUSE and TVSE, TVSE required longer operation time, possibly due to the necessity of intracorporeal suturing and anastomosis took longer [21]. Further study with a larger population is warranted to investigate the effects of TASE in the overall operation time in SPLS.

A drawback to TASE is its limited application; it can be applied to the patients with a small tumor, i.e. a tumor diameter of 5 cm or less in this study. We have designed anal trocars in various sizes up to 6 cm (3-, 4-, 5-, and 6-cm) for the restoration of rectal sphincter muscle tone. The median tumor diameter in the TASE group was 3.0 cm (2.0–5.0 cm) in this study. However, we think as surveillance system enables the early detection of colorectal cancer, the inclusion of patients who would benefit from TASE would be wider.

In our series, we did not observe fecal incontinence or any complications related to anorectal function. Excessive pressure during TASE can induce fecal incontinence, possibly due to the loss of anal sphincter muscle tone [12]. Therefore, in every surgery, we attempted to avoid excessive rectal dilatation. We gently retrieved the specimen by way of the metallic anal trocar made of stainless steel, which gradually dilated the anus and rectal wall within very limited time span. Multiple reports on the anorectal function after trananal endoscopic microsurgery (TEM) has shown that TEM, even repeated TEMs, does not affect anal sphincter pressure, rectoanal reflexes, rectal sensation or compliance [22–26]. Thus we think TASE is safe, in terms of anorectal function, in patients with a tumor diameter of ≤5 cm.

The limitations of this study are those common to all database research. As a retrospective review of prospectively collective data, our results should be confirmed by a prospective trial. Next, the limitations of this pilot study also include the small patient population, especially TASE patients (n = 15). In addition, TUSE and TASE groups seemed to be not completely balanced; TUSE patients showed higher incidences of T3 tumor (61.8% vs. 20.0%, *P* = 0.013) and lymphovascular invasion (86.8% vs. 53.3%, *P* = 0.007), suggesting advanced histology.

## Conclusion

This pilot study shows that with the exception of operation time, surgical outcomes of TASE were comparable to those of TUSE. Even though TASE prolonged operation time, it appeared to decrease over time, suggesting an adequate learning curve. In addition, TASE procedure did not affect anorectal function. In the SPLS, extension of a single incision for the purpose of specimen extraction can undermine the merits of SPLS either by hurting cosmesis or by increasing wound morbidity, such as umbilical hernia. Though the establishment of the safety and feasibility of TASE requires further study, the implement of TASE in SPLS is expected to provide one way of reducing wound-related complications in patients with a tumor diameter of ≤5 cm.
